# *In situ* microseismicity reveals lithospheric accretion at the ultraslow-spreading Gakkel Ridge, Arctic Ocean

**DOI:** 10.1093/nsr/nwag034

**Published:** 2026-01-19

**Authors:** Zhiteng Yu, Jiabiao Li, Weiwei Ding, Yinxia Fang, Tao Zhang, Fansheng Kong, Yan Jia, Xiongwei Niu, Pingchuan Tan, Zhangju Liu, Zhezhe Lu

**Affiliations:** State Key Laboratory of Submarine Geoscience, Second Institute of Oceanography, Ministry of Natural Resources, China; State Key Laboratory of Submarine Geoscience, Second Institute of Oceanography, Ministry of Natural Resources, China; State Key Laboratory of Submarine Geoscience, Second Institute of Oceanography, Ministry of Natural Resources, China; State Key Laboratory of Submarine Geoscience, Second Institute of Oceanography, Ministry of Natural Resources, China; State Key Laboratory of Submarine Geoscience, Second Institute of Oceanography, Ministry of Natural Resources, China; State Key Laboratory of Submarine Geoscience, Second Institute of Oceanography, Ministry of Natural Resources, China; State Key Laboratory of Submarine Geoscience, Second Institute of Oceanography, Ministry of Natural Resources, China; State Key Laboratory of Submarine Geoscience, Second Institute of Oceanography, Ministry of Natural Resources, China; State Key Laboratory of Submarine Geoscience, Second Institute of Oceanography, Ministry of Natural Resources, China; Southern Marine Science and Engineering Guangdong Laboratory (Guangzhou), China; State Key Laboratory of Submarine Geoscience, Second Institute of Oceanography, Ministry of Natural Resources, China; State Key Laboratory of Submarine Geoscience, Second Institute of Oceanography, Ministry of Natural Resources, China

## Abstract

New on-site microseismic data reveal a deep-seated fault at the Gakkel Ridge in the Arctic Ocean, which is likely linked to an incipient detachment fault that controls the ridge’s segmentation.

Oceanic lithospheric accretion at mid-ocean ridges is a fundamental process in plate tectonics, yet its manifestation at ultraslow-spreading ridges (full spreading rates of <20 mm/year) remains insufficiently constrained. The Gakkel Ridge in the Arctic Ocean, as the most magma-starved ridge and the Earth’s slowest spreading endmember (<15 mm/year), is expected to have a thick lithosphere [1]. Notably, unlike faster slow-spreading mid-ocean ridges, the Gakkel Ridge lacks major transform faults that offset its spreading centers, but it exhibits distinct ridge regimes [2], including a western volcanic zone, a central sparsely magmatic zone and an eastern volcanic zone [2] (Fig. 1a). Although large amounts of fresh abyssal peridotites have been recovered from the sparsely magmatic zone of the Gakkel Ridge [2], large-scale oceanic detachment faults, which have been long considered as the presumed driver for mantle rock exhumation [3], have not been observed at the Gakkel Ridge. This discrepancy raises fundamental questions about its spreading mechanisms and segmentation processes.

**Figure 1.**
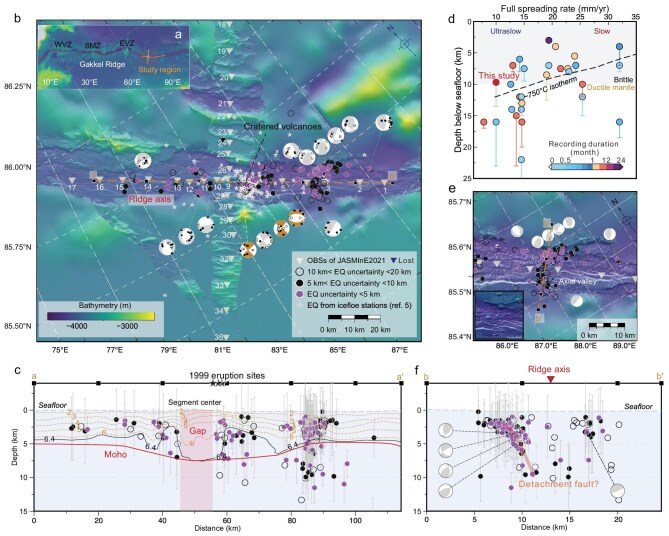
Study area and microseismicity. (a) The inset shows the bathymetric map of the Gakkel Ridge. WVZ = Western volcanic zone; SMZ = sparsely magmatic zone; EVZ = Eastern volcanic zone [2]. (b) Bathymetry of the 85.0°E/85.6°N volcanic center at the Gakkel Ridge, showing microearthquakes determined in this study. The colored circles indicate various location uncertainties (refer to the legend for details). High-quality focal mechanism solutions are in brown, and gray ones are of lower quality, with P-wave polarities plotted for reference. The gray triangles show the deployed OBSs, with OBS6 (lost) marked in blue. The gray stars are microearthquakes recorded by ice floe seismic stations [5]. The black stars denote three reported cratered volcanoes. (c) Earthquake depths along the ridge axis [dashed brown line aa′ in a]. The gray bars denote the depth error estimates derived from bootstrap analysis. The black squares at the top mark the 20 km intervals. The P-wave velocity intervals and the Moho interface were derived from a seismic refraction profile [7]. (d) Statistical analysis of the maximum depth distribution of microseismicity and full spreading rates (Table S2). The colored circles denote the depths of earthquakes at slow- and ultraslow-spreading ridges, with the color scale indicating the observation duration (months). The dashed black line indicates the 750°C isotherm. (e) Microseismicity at 88°E near the ridge segment end. The inset map shows the bathymetry without seismicity. (f) A cross-section of earthquakes across the ridge axis, whose location (bb′) is shown in (e).

Microseismicity provides critical constraints on oceanic lithospheric structure, as the maximum depth of microearthquakes acts as a proxy for the brittle lithospheric thickness and the depth of the brittle–ductile transition zone [1,4]. However, such investigations are notably rare at the

Gakkel Ridge due to its persistent ice cover. Prior microseismicity studies at the 85°E volcanic center of the Gakkel Ridge were conducted using seismic stations installed on floating ice [5] (Fig. 1a), which not only prevented S-wave recordings but also posed significant challenges in accurately locating earthquakes due to continuous station drift. Here we report *in situ* seismic observations from ocean bottom seismographs (OBSs) deployed at the eastern Gakkel Ridge (78°–90°E) during the 2021 JASMInE expedition (Fig. 1). By analyzing the spatial distribution of microseismicity and stress patterns, we image a thin base of brittle lithosphere along the Gakkel Ridge axis and present evidence for a deep-rooted fault at the segment end that may be linked to an incipient detachment fault, which substantially influences hydrothermal circulation and ridge segmentation. These results provide new insights into lithospheric accretion modes of the global mid-ocean ridge system.

Our study region lies in the eastern volcanic zone of the Gakkel Ridge in the Arctic Ocean, characterized by a full spreading rate of ∼11 mm/year and a spreading direction nearly orthogonal to the ridge axis (Fig. 1a). The bathymetric data reveal a large volcanic center (∼13 km × 5 km) situated at 85°E, with water depths of ∼3700–4000 m (Fig. 1b). The 85°E volcanic center experienced an explosive submarine eruption in 1999 [6],

with three cratered volcanoes observed on the axial valley (Fig. 1b), indicating a high volatile concentration in the magma system here. The seismic refraction profiles indicate that crustal thickness varies significantly, with 7.5 km at the segment center (85°E) compared to ∼3.5 km at the segment end (88°E) [7].

During the 2021 JASMInE cruise, we deployed 17 and 18 OBSs along and across the 85°E volcanic center, respectively, with instrument spacing of 5–10 km (Fig. 1b). These OBSs continuously recorded seismicity for up to 22 days (averaging 13 days), including ∼4 days of air-gun shooting. We located 234 microearthquakes (∼0.2 ≤ M_L_ ≤ 3.0) along the ridge axis (Fig. 1b). The detailed description of the methods is available in the Supplementary data (Figs S1–S12, Tables S1 and S2). For approximately 64% of the events, the hypocentral uncertainties are less than 10 km, and 56% of the events have depth uncertainties less than 5 km (Fig. 1c). The majority of events occurred between 2 and 8 km depth, with some reaching down to 10 km below the seafloor (bsf) (Fig. 1c). Most focal mechanisms here reveal normal faulting, consistent with the extensional stress expected at the mid-ocean ridge systems. However, three strike-slip focal mechanisms identified beneath the 1999 eruption sites (Fig. 1b) likely reflect shear faulting driven by dike propagation or melt intrusion in the crust. While the strike-slip stress patterns indicated by earthquake swarms are well documented in volcanic systems (e.g. Mayotte volcano, Indian Ocean [8]), our short observation duration (<1 month) likely prevented the capture of a sufficient number of such events. However, recent magnetotelluric data reveal a low-resistivity zone at the 85°E volcanic center [9], indicating that significant magmatic processes sustain geodynamic activity at the 85°E volcanic center, even 20 years after the submarine eruption. Longer-duration observations are required to fully capture this magmatism-related seismicity.

A seismic gap exists beneath the 85°E volcanic center, delineated by neighboring earthquakes down to 8 km bsf (Fig. 1c). Scattered microseismicity occurs beneath the 1999 explosive volcano eruption sites (Fig. 1b and c). The aseismic nature of the volcanic center at 85°E (Fig. 1c) mimics features found at other ultraslow-spreading ridges, such as the Southwest Indian Ridge (SWIR) and the Knipovich Ridge [1,4]; this aseismic behavior is interpreted to reflect elevated temperatures beneath the volcanic center that inhibit brittle failure. Previous studies report that, within ∼30 km of the volcanic center, the axial brittle lithosphere thickens rapidly to depths of ∼20 km due to the enhanced conductive cooling and hydrothermal circulation [1,4]. However, our observed seismicity along the studied 100 km-long portion of the Gakkel Ridge is much shallower, with the maximum earthquake depth not exceeding ∼10 km bsf (Fig. 1c). The results indicate a thin base of brittle lithosphere, with no significant shallowing from the volcanic center to the segment end over a short distance, as observed at the SWIR and the Knipovich Ridge [1,4]. This depth range is substantially shallower than the 20 km reported at other ultraslow-spreading ridges [1,4], but it is consistent with thermal models predicting the depth of the 750°C isotherm under ultraslow-spreading conditions [10] (Fig. 1d). We propose that the lithosphere in this region is thinned by the large, hot volcano at 85°E, which induces extensive thermal weakening; importantly, this thermal anomaly associated with the 85°E volcanic center influences lithospheric rheology to an extent far beyond previous estimates of <30 km, reaching ∼70 km from the segment center. In addition, thick sediment layers in the axial valley [7] would blanket the seafloor, impeding efficient heat loss through hydrothermal cooling, which contributes to the development of a thin lithosphere from the segment center to its end.

Unexpectedly, the majority of the seismicity is located at 88°E near the segment end, where no volcanic cones or other volcanic features are observed in the bathymetric data (Fig. 1e). Similar earthquake swarms at the ultraslow-spreading SWIR segment 8 were interpreted as dike intrusions from the horizontal movement of the magma chamber at the segment center [1]. However, our calculated b-value of ∼1.0 (Fig. S9) is lower than the reported b-value of ∼1.5 during the 1999 volcanic activity at the 85°E segment center [6]. This relatively low b-value likely reflects a shift in the local stress regime and melt conditions compared to the 1999 episode. The observed normal b-value (∼1.0) indicates a seismic regime governed by the regional tectonic stress and reduced melt supply, in contrast to the high b-value (>1.5) characteristic of magmatically driven processes and elevated pore-fluid pressures [6]. In addition, spectrogram analysis of seismic waveforms reveals a broad frequency distribution (2–15 Hz) for tectonic events (Fig. S10), excluding the possibility of dike intrusions, which are typically characterized by low-frequency signals (usually <5 Hz). Thus, we propose that these earthquakes are tectonic in nature rather than volcanic.

Our observed earthquakes reveal a steeply dipping fault plane (∼70°) oriented NE–SW. Unlike normal faults confined to the crust, this fault is deeply rooted, cutting the entire 5 km-thick crust, extending into the upper mantle, and reaching a depth of 10 km bsf (Fig. 1f). This observation indicates a thicker brittle lithosphere at the segment end compared to that at the segment center, which is consistent with the reported significant along-axis lithospheric thickness variations from a magnetotelluric study [9]. The seismicity pattern here reveals highly asymmetric spreading, with most events clustered at the northern edge of the axial valley and only sparse seismicity along the axial ridge axis (Fig. 1e). Although focal mechanisms are not well constrained, our results document both compressional and extensional stresses, suggestive of a complex faulting system (Fig. 1e and f). This diverse stress pattern is characteristic of a detachment fault system, which typically exhibits normal faults in the footwall and reverse faults in response to fault bending [11]. Taken together, the deep seismicity, asymmetric spreading and diverse stress pattern suggest the possible presence of an underlying detachment fault in this area (Fig. 1f). This interpretation is supported by previous microseismicity studies, which show that detachment faults are typically deeply rooted [10–12] and associated with asymmetric spreading [11,12]. However, the absence of a corrugated surface in the bathymetric data (Fig. 1e, Fig. S12), a key characteristic of detachment faults [3], suggests that the identified fault is currently an incipient detachment fault or a deep shear zone that has not yet matured sufficiently to develop the corrugated topography. This interpretation is consistent with ‘young’ detachment faults observed at the Trans-Atlantic Geotraverse (TAG) (Mid-Atlantic Ridge) [12] and Loki’s Castle (Mohns–Knipovich Ridge) [13] hydrothermal vents, which similarly lack corrugated surfaces but present deep-seated activity. At the TAG detachment fault, the emplacement of lower crustal and mantle materials produced significant P-wave velocity anomalies (>0.5–2 km/s) in the shallow crust [12]. In contrast, our study region shows smaller elevated P-wave velocity anomalies (∼0.5–1.0 km/s) (Fig. 1c), suggesting that the emplacement of high-velocity material is less pervasive here. In addition, the identified fault does not roll over from a steep angle to a lower angle (Fig. S12), consistent with the ‘young’ detachment fault at Loki’s Castle hydrothermal vent [13]. Therefore, we conclude that the inferred fault is an incipient detachment fault or a deep shear zone that has the potential to evolve into a fully developed oceanic core complex and a typical detachment fault system.

The deep-rooted fault would introduce substantial water into the mantle, leading to extensive peridotite serpentinization in the uppermost mantle that coincides with low densities indicated by gravity data [7]. The extensive deep hydration channeled through the fault plane would further deepen the lithosphere–asthenosphere boundary, which in turn suppresses mantle melting underneath [9]. The deep-rooted fault system likely controls ridge segmentation by establishing strong rheological boundaries. Given the absence of large-offset transform fault systems at the Gakkel Ridge, the deep faulting system plays a more important role in ridge segmentation. Notably, the deep shear zone/detachment fault can act as an effective conduit for hydrothermal circulation, implying potential sites of active hydrothermal vents. Previous studies have detected hydrothermal fluids at 69°E [14] and hydrothermal sediments at 96°E [15] along the Gakkel Ridge, where volcanic centers are absent, similar to our study region at 88°E, and these hydrothermal signals were interpreted as originating from adjacent volcanic centers (85°E and 92°E). However, an alternative explanation is that the deep-rooted faults at segment ends may have developed to serve as effective conduits for deep hydrothermal circulation. The spatial correlation with vent activity requires further detailed investigation for validation.

Our microseismic observation indicates a relatively hot lithospheric regime, consistent with the thick crust determined from seismic refraction profiles [7], and challenges the previously suggested cold and thickened lithosphere [1]. Axial thermal influence from the segment center likely facilitates localized melting at the segment end, enabling the formation of deep detachment faults there. It is noted that the short duration of our dataset provides only a snapshot of microseismicity in this region. Future studies require a long-duration seismic network for passive signal recording, which is critical for studying the lithospheric accretion at the Gakkel Ridge.

## Supplementary Material

nwag034_Supplemental_File

## References

[1] Schlindwein V, Schmid F. Nature 2016; 535: 276–9. 10.1038/nature18277 27362231

[2] Michael PJ, Langmuir CH, Dick HJB et al. Nature 2003; 423: 956–61. 10.1038/nature01704 12827193

[3] MacLeod CJ, Searle RC, Murton BJ et al. Earth Planet Sci Lett 2009; 287: 333–44. 10.1016/j.epsl.2009.08.016

[4] Meier M, Schlindwein V, Scholz J et al. Geochem Geophys Geosyst 2021; 22: e2020GC009375. 10.1029/2020GC009375

[5] Korger EIM, Schlindwein V. Geophys J Int 2014; 196: 539–51. 10.1093/gji/ggt390

[6] Tolstoy M, Bohnenstiehl DR, Edwards MH et al. Geology 2001; 29: 1139. 10.1130/0091-7613(2001)029<1139:SCOVAA>2.0.CO;2

[7] Zhang T, Li J, Niu X et al. Nature 2024; 633: 109–13. 10.1038/s41586-024-07831-0 39169191 PMC11374676

[8] Feuillet N, Jorry S, Crawford WC et al. Nat Geosci 2021; 14: 787–95. 10.1038/s41561-021-00809-x

[9] Zhang T, Li J, Ding W et al. Natl Sci Rev 2025; 12: nwaf077. 10.1093/nsr/nwaf077 40260320 PMC12010957

[10] Grevemeyer I, Hayman NW, Lange D et al. Geology 2019; 47: 1069–73. 10.1130/G46577.1

[11] Parnell-Turner R, Sohn RA, Peirce C et al. Geology 2017; 45: 923–6. 10.1130/G39232.1

[12] deMartin BJ, Sohn RA, Pablo Canales J et al. Geology 2007; 35: 711–4. 10.1130/G23718A.1

[13] Pilot M, Lien MJ, Schlindwein V et al. Geochem Geophys Geosyst 2024; 25: e2024GC011732. 10.1029/2024GC011732

[14] Baker ET, Edmonds HN, Michael PJ et al. Geochem Geophys Geosyst 2004; 5: eQ08002. 10.1029/2004GC000712

[15] Xue W, Wang Y, Qiu Z et al. J Ocean Limnol 2025; (in press). 10.1007/s00343-025-5112-2

