# Supplementary material for: In situ microseismicity reveals lithospheric accretion at the ultraslow-spreading Gakkel Ridge, Arctic Ocean
Source: Natl Sci Rev. 2026 Jan 19;13(4):nwag034. doi: 10.1093/nsr/nwag034 (PMC12892356; doi:10.1093/nsr/nwag034)
Supplement: nwag034_Supplemental_File [file nwag034_supplemental_file.pdf]

Supplementary Information for
In situ microseismicity reveals lithospheric accretion at the ultraslow-spreading Gakkel Ridge, Arctic Ocean

Zhiteng Yu[1], Jiabiao Li[1,2*], Weiwei Ding[1,2], Yinxia Fang[1], Tao Zhang[1], Fansheng Kong[1], Yan Jia[1], Xiongwei Niu[1], Pingchuan Tan[1], Zhangju Liu[1], Zhezhe Lu[1]

[1]*State Key Laboratory of Submarine Geoscience, Second Institute of Oceanography, 310012 Hangzhou, China*

[2]*School of Oceanography, Shanghai Jiao Tong University, Shanghai, China*

[*]Corresponding author: Jiabiao Li (jbli@sio.org.cn)

This PDF file includes:

Supplementary Methods

Supplementary Figures S1-S12

Supplementary Tables 1-2

References used in this document

Supplementary Methods

Seismic data acquisition

In August 2021, a total of 43 ocean bottom seismographs (OBSs, Nos. 1–44) were deployed along the eastern Gakkel Ridge (Fig. 1a), with the aim of recording microearthquakes as part of an active-source seismic experiment (JASMInE) [1]. The instruments were spaced at an interval of ~5-10 km, and OBS6 was lost during the deployment. Among the remaining OBSs, eight OBSs (Nos. 36–44) deployed at 92–100°E were deployed for only one to three days (Fig. 1a). Also, those OBSs were arranged in a straight line, and not deployed with other instruments simultaneously, leading to insufficient time to record microearthquakes and unsuitable for earthquake location. In this study, we selected 35 OBSs (Nos. 1–35) deployed in the 85°E segment; however, due to the loss of OBS6, the final dataset consists of 34 instruments (Fig. 1b). 17 OBSs (Nos. 1–17) were deployed along the Gakkel Ridge axis (78°E-90°E), covering a distance of ~120 km, while 18 OBSs (Nos. 18–35) were deployed across the 85°E volcanoes, covering a distance of ~110 km (Fig. 1b). The average recording time is ~13 days, with a maximum of ~22 days.

Arrival detection

Due to the large noise caused by the presence of the air-gun source signals on the waveforms, traditional phase-picking trigger methods, such as the short-term average/long-term average (STA/LTA) algorithm, did not perform effectively in this study. We applied an attentive deep-learning model for earthquake detection and phase picking by identifying the earthquake waveforms within the OBSTransformer package [2] based on the EQTransformer picker [3]. This deep-learning model is specifically trained using a large dataset of seismic data collected from 11 temporary OBS arrays worldwide [2]. We used threshold values of 0.5 for detection and phase picking, respectively. As a result, we identified and associated 964 earthquakes, e with each supported by at least three seismic phase picks, including either P-wave or S-wave picks. Then we manually checked the event to ensure the picked phases were correct (Fig. S1). The P-wave and S-wave phase-pick errors were 0.1 s and 0.2 s, respectively. The OBSTransformer package shows good performance even during the airgun shooting time (Fig. S2).

One-dimensional reference velocity model

We constructed nine one-dimensional (1-D) P-wave velocity models (Fig. S3a). These models were derived from a wide-angle seismic refraction profile along the studied ridge axis [4], which offers velocity constraints at depths down to ~11 km below sea level (Fig. S3b). The nine 1-D velocity models are located linearly along the ridge axis, extending from the segment center to the segment end [4]. The S-wave velocity model was estimated by using a constant Vp/Vs ratio of ~1.6, which was determined from a Wadati diagram (Fig. S4). The 1-D model was chosen for the subsequent earthquake location and focal mechanism computation, based on the optimal combination of a high number of located events, a low average root-mean-square (RMS) residual, and minimal horizontal and depth uncertainties (Fig. S3c). We found that the 1-D velocity model close to the segment end (Model 9 in Fig. S3b) was the best-fitting model (Fig. S3c). In addition, we removed the S-wave delays, which are caused by very low velocities in unconsolidated sediments [5], by identifying the P-s conversion phases on the horizontal components [6]. The S-wave delays range from 0.3 s to 1.3 s in this study.

Hypocenter location

Earthquake hypocenters were calculated using the nonlinear oct-tree search algorithm implemented in the NonLinLoc program [7]. The maximum likelihood solution was selected as the preferred hypocenter, with the one-dimensional error ellipsoid (68% confidence) estimated from the posterior density function scatter samples [7]. Iterative calculations of station corrections (Fig. S3c) were applied to remove the one-dimensional velocity effects until the average RMS misfit reached a minimum. We located 243 local microearthquakes, each of which has more than five arrivals. The earthquake occurred continuously during the OBS deployment time (Fig. S2).

Microearthquakes mainly occurred in the segment end at 88°E, where their uncertainties are relatively large (Fig. 1c). We found that axial OBSs (No. 1–17) recorded nearly twice the number of arrival picks compared to off-axis OBSs (No. 18–34) (Fig. S5). This observed discrepancy is likely due to high energy attenuation of seismic waves caused by thick sediments in the study region [1]. Consequently, distant deployed off-axis OBSs (>70 km) failed to detect clustered events at ~88°E with insufficient sensitivity, leading to relatively low location accuracy for these events. We analyzed all the tested 1-D velocity models, and the results

indicated a depth range not exceeding ~12 km (Fig. S6).

A double-difference earthquake relocation algorithm [8] was used to improve the hypocenter accuracy for the 135 earthquakes determined on more than six OBSs, with RMS residuals of <0.25 s and azimuthal gaps of <270° (Fig. S7). A minimum of eight catalog links per event pair was required to form a continuous cluster, and the cross correlation is set to be >0.6. The results show that the focal depth shifted a little, indicating stable earthquake locations (Fig. S7). The earthquake relocations will be replaced in the final catalog.

We further used bootstrap analysis [9,10] to verify the robustness of these earthquake relocations. We randomly resampled the seismic phases of each event 200 times. Due to the nature of resampling with replacement, some phases might be selected multiple times, while others might not be selected at all. To simulate arrival-time picking errors, random noise ranging from −0.1 to +0.1 s and from −0.2 to +0.2 s was added to the synthetic travel times of P-waves and S-waves, respectively. We then relocated the earthquakes using the 200 resampled datasets. In this way, we obtained 200 estimates of the location uncertainties of the earthquakes. The deviations of the relocated positions relative to the initial location basically follow a normal distribution, with the 95% confidence intervals of uncertainties being 3.89 km, 2.82 km, and 3.29 km in north-south, east-west, and vertical directions, respectively (Fig. S8). In addition, we calculated the relative uncertainties for each event via bootstrap sampling, which were defined as the average deviations from the initial locations. These uncertainties were then used to replace the original ones (Fig. 1c).

Local magnitude

Earthquake magnitudes were determined using the local magnitude M_L scale [11]:

$$M_L = logA + 1.11\ log\ (D) + 0.00189\ D - 2.09,$$

where A is the maximum amplitude (in millimeters) measured on synthetic Wood-Anderson seismograms generated using the Seismic Analysis Code software [12], and D is the hypocentral distance (in kilometers). The b-value is calculated to be ~1.0 (Fig. S9), indicating typical tectonic events. The spectrogram analysis (Fig. S10) reveals that the earthquakes at 88°E exhibit a broad frequency range (~2-15 Hz), suggestive of typical characteristics of tectonic earthquakes rather than the low-frequency seismic signals associated with volcanic

activities.

First-motion focal mechanisms

We used the SKHASH Python package [13] to compute focal mechanisms based on P-phase first-motion polarities manually identified from unfiltered vertical-component waveforms. A total of 15 acceptable focal mechanism solutions were obtained (Fig. S11) by applying rigorous selection criteria following well-established community standards [14]: ≥8 P-wave polarities, an azimuthal gap of <180°, a take-off angle gap of <60°, an RMS fault plane uncertainty of <50°, an average misfit of <30%, and a station distribution ratio of >0.5 (Table S1). Three solutions met the category **B** classification under the HASH criteria [14], indicating high-confidence focal mechanisms with well-constrained fault planes (Fig. S11).

The maximum depth of earthquakes

Following the method of ref. [15], we compiled the maximum depth of earthquakes along the ridge axis documented to date at slow- and ultraslow-spreading ridges around the world, as well as the full spreading rate and recording duration on each site (Fig. 1d, Table S2). In this study, we selected the maximum depths that were well constrained by several earthquakes instead of only one event to avoid bias in the location process (Table S2). The Rainbow massif is located at a non-transform discontinuity, which is also included in the plot for reference (Fig. 1d, Table S2).

Supplementary Figures

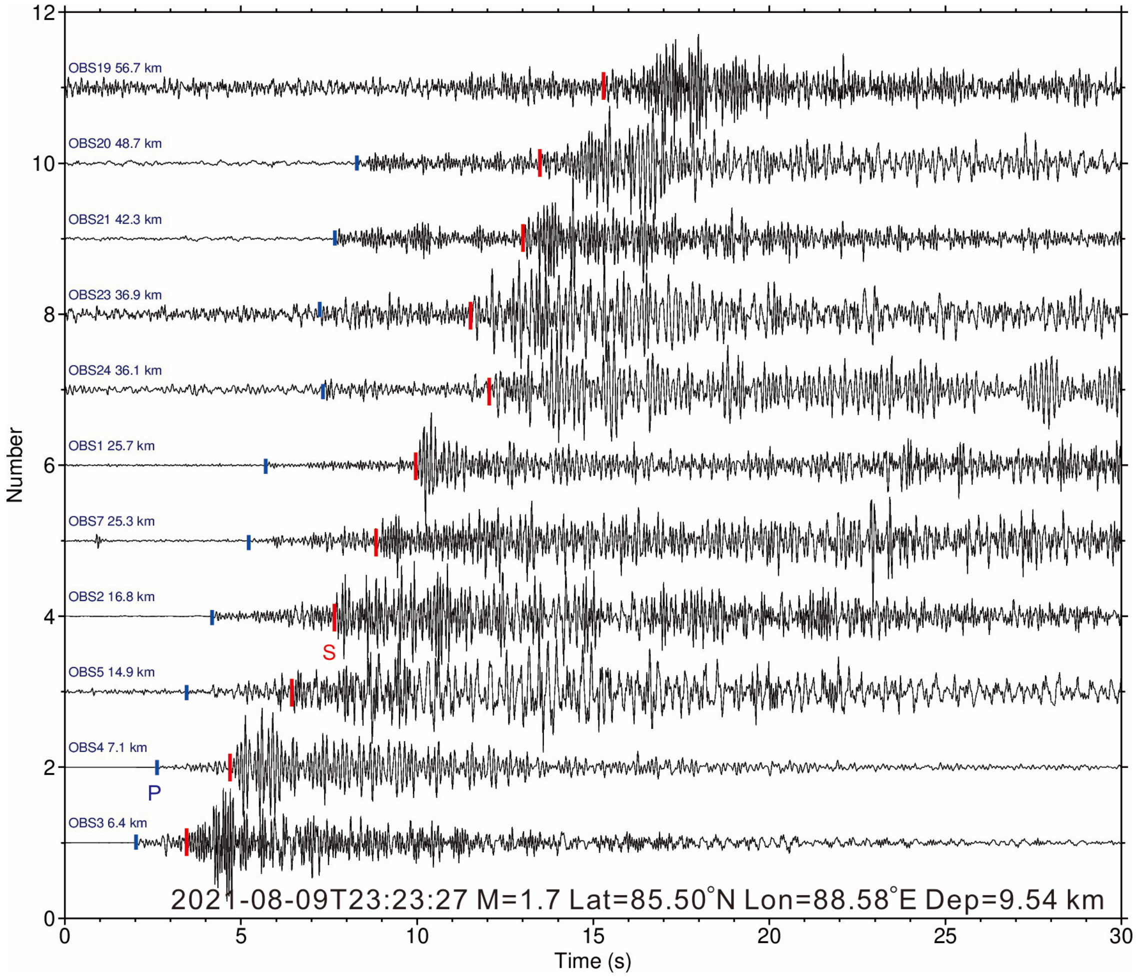

Figure S1. Example seismograms of a microearthquake obtained in this study. The hypocentral distance for each station is marked on the left. The blue and red bars denote the picked P-wave and S-wave phases, respectively.

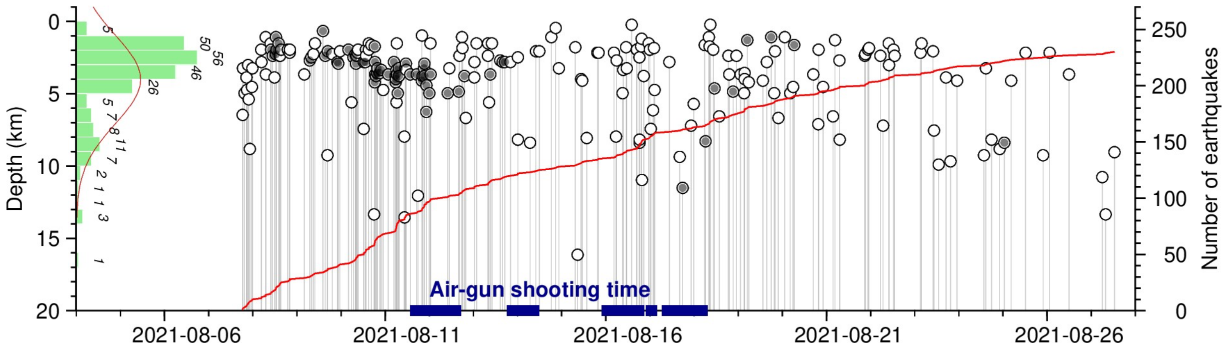

Figure S2. The distribution of earthquake focal depths as a function of dates from August 6 to August 28, 2021. The red line indicates the cumulative number of earthquakes. The gray circles represent the earthquakes clustered at 88°E. The air-gun shooting time is marked in bold blue lines. Depth histograms are shown on the left, with a red line showing the normal distribution curve.

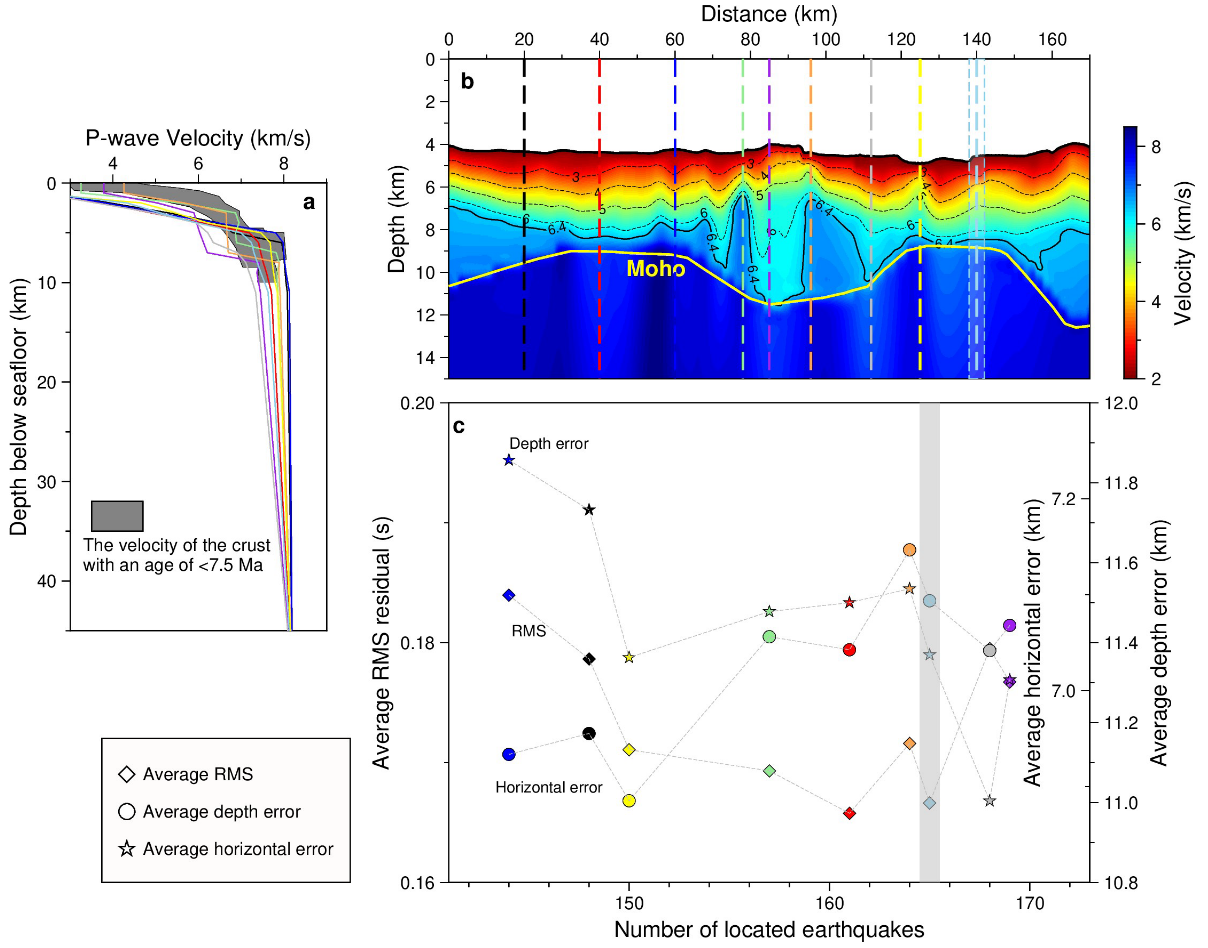

Figure S3. One-dimensional (1-D) P-wave velocity models and earthquake locations. (**a**) Nine 1-D velocity models were derived from an active-source seismic refraction profile [4]. The gray shades indicate the velocity of an age of <7.5 Ma [16]. (**b**) The two-dimensional P-wave velocity model along the Gakkel Ridge axis is presented. The dashed colored lines show nine

1-D velocity models in (**a**). The Moho interface is marked by a yellow line. (**c**) Average RMS residuals (diamonds), depth uncertainties (stars), and horizontal uncertainties (circles) are plotted as a function of the number of located earthquakes. The gray shade shows the selected model for earthquake location.

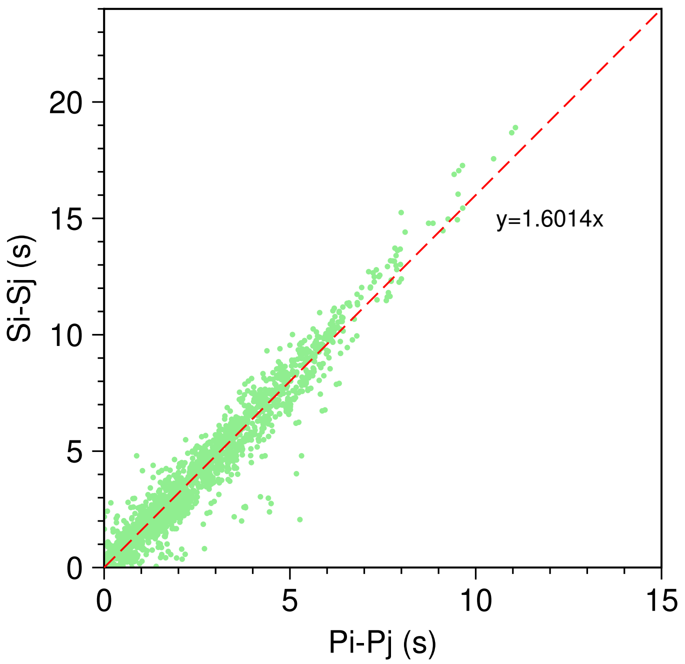

Figure S4. Wadati diagram. Green dots represent the time differences between P-arrivals (Pi-Pj) versus those between S-arrivals (Si-Sj) for each station pair (i, j) of each event, yielding a Vp/Vs ratio of ~1.6.

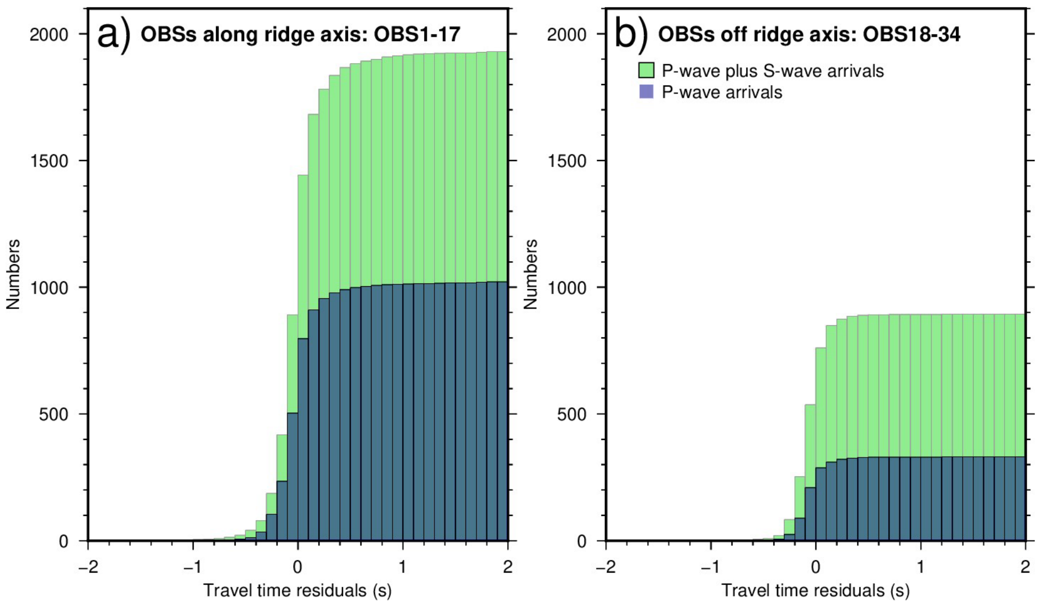

Figure S5. Travel time residuals and numbers of arrivals for OBSs located along (**a**) and across (**b**) the Gakkel Ridge axis. The dark blue columns indicate P-wave arrivals, while the green ones denote both P-wave and S-wave arrivals.

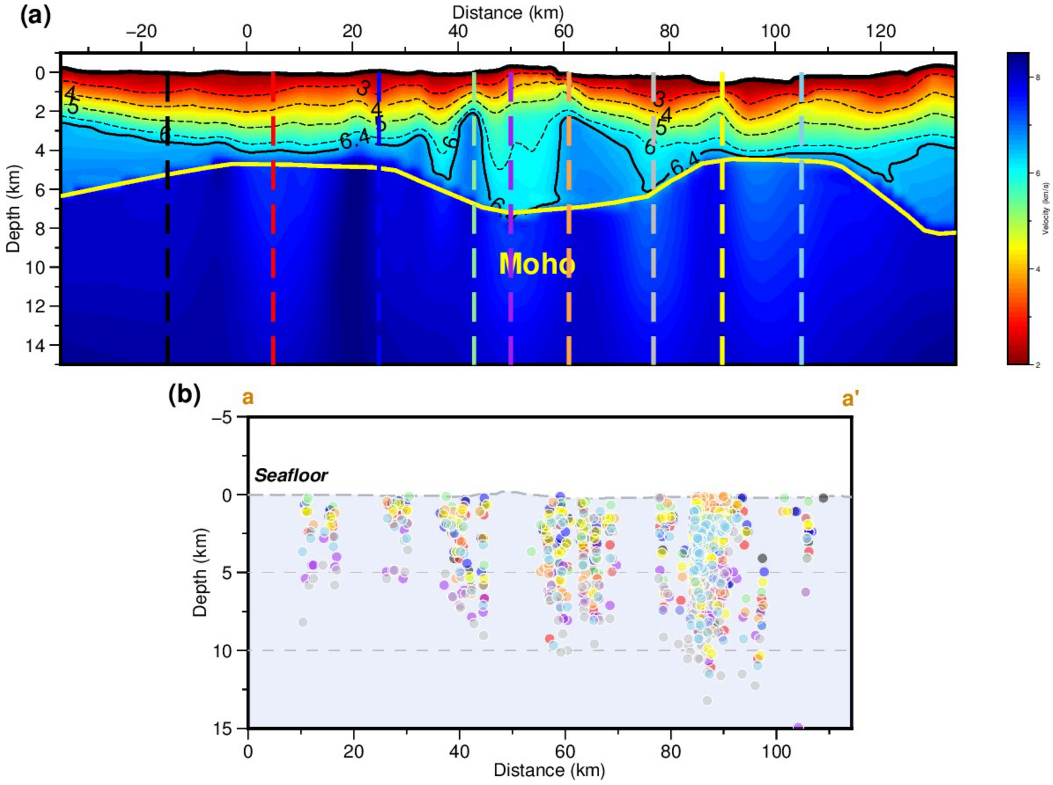

Figure S6. Located earthquake depths using all tested 1-D P-wave velocity models. (**a**) The seismic refraction profile used in this study. Dashed colored lines indicate nine 1-D velocity models. (**b**) Hypocentral depths along the ridge axis using different 1-D velocity models (colored circles).

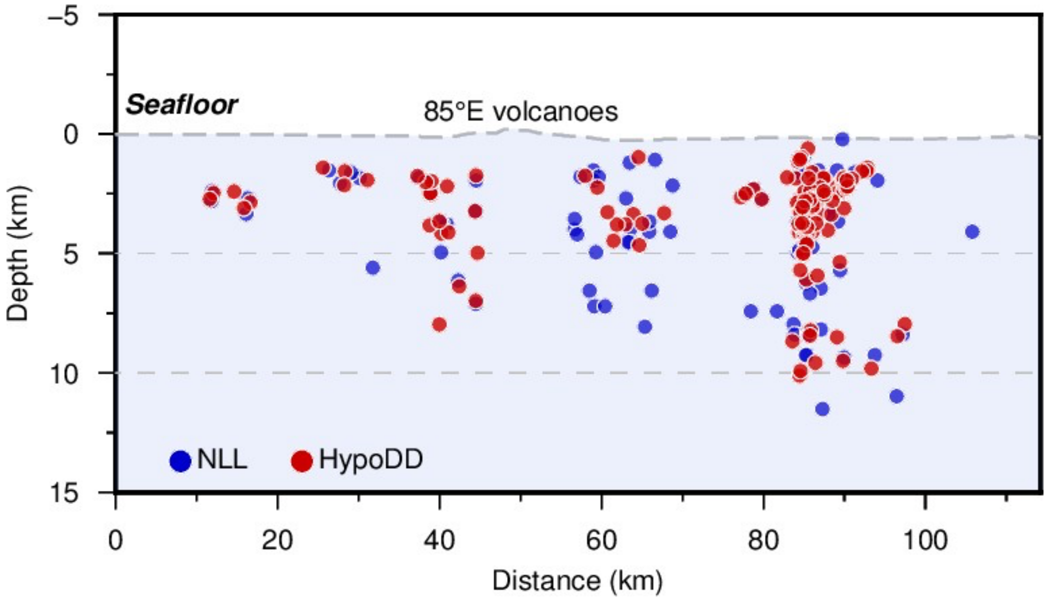

Figure S7. The double difference relocation hypocentral depths along the ridge axis. The blue and red dots show the initial locations by the NonLinLoc program [7] and relocation results by the HypoDD program [8], respectively.

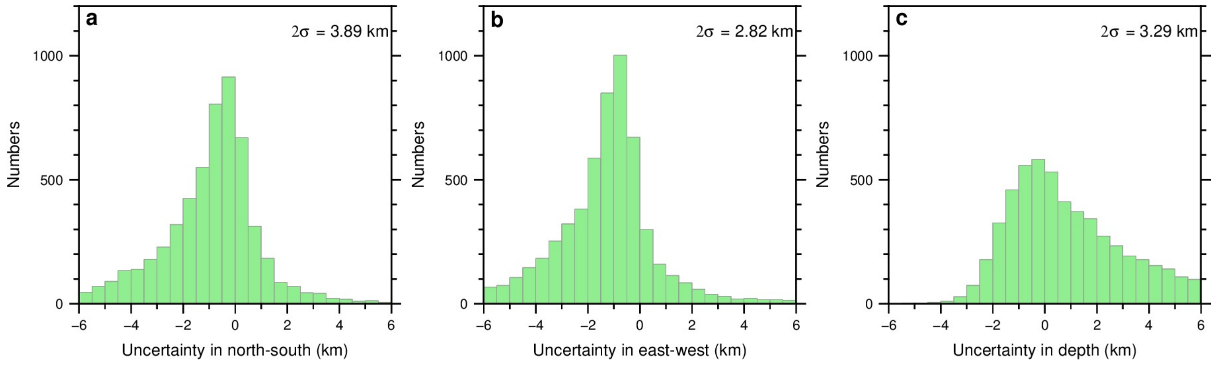

Figure S8. The bootstrap estimates of uncertainties in north-south (**a**), east-west (**b**) and vertical directions (**c**), respectively. The twice of standard deviation (2σ) of location errors are shown on the right corner.

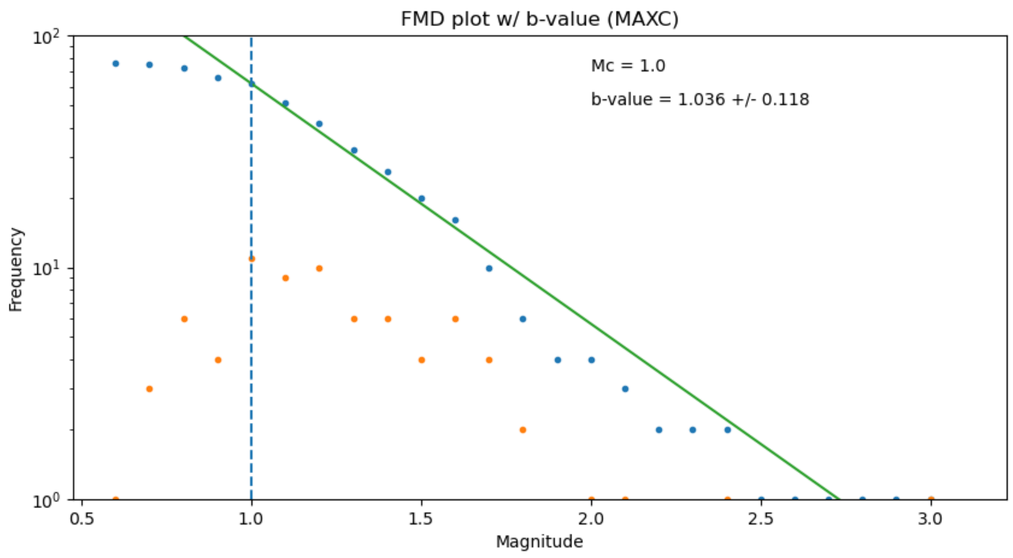

Figure S9. Magnitude completeness and b-value were obtained using the earthquake catalog in this study.

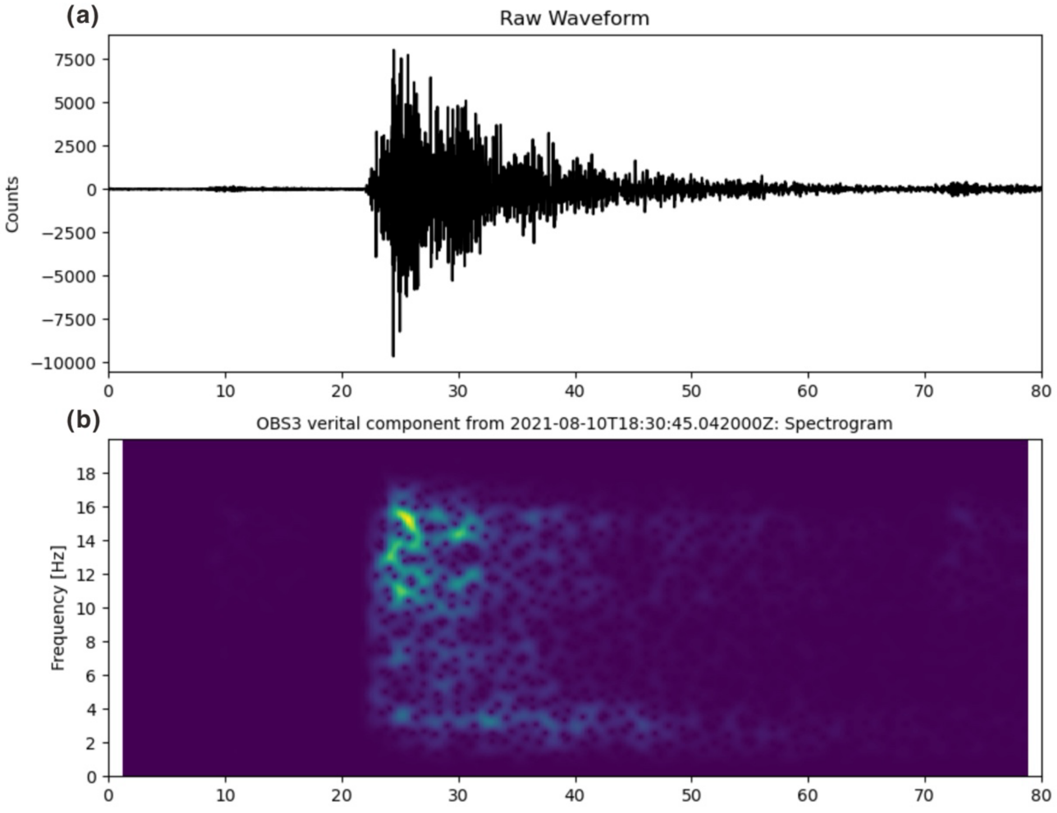

Figure S10. (**a**) The waveform of one example earthquake recorded at OBS3 shown at the top. (**b**) The spectrogram plot of the vertical component. The starting time is shown at the top, and the horizontal axis indicates the recording time in seconds.

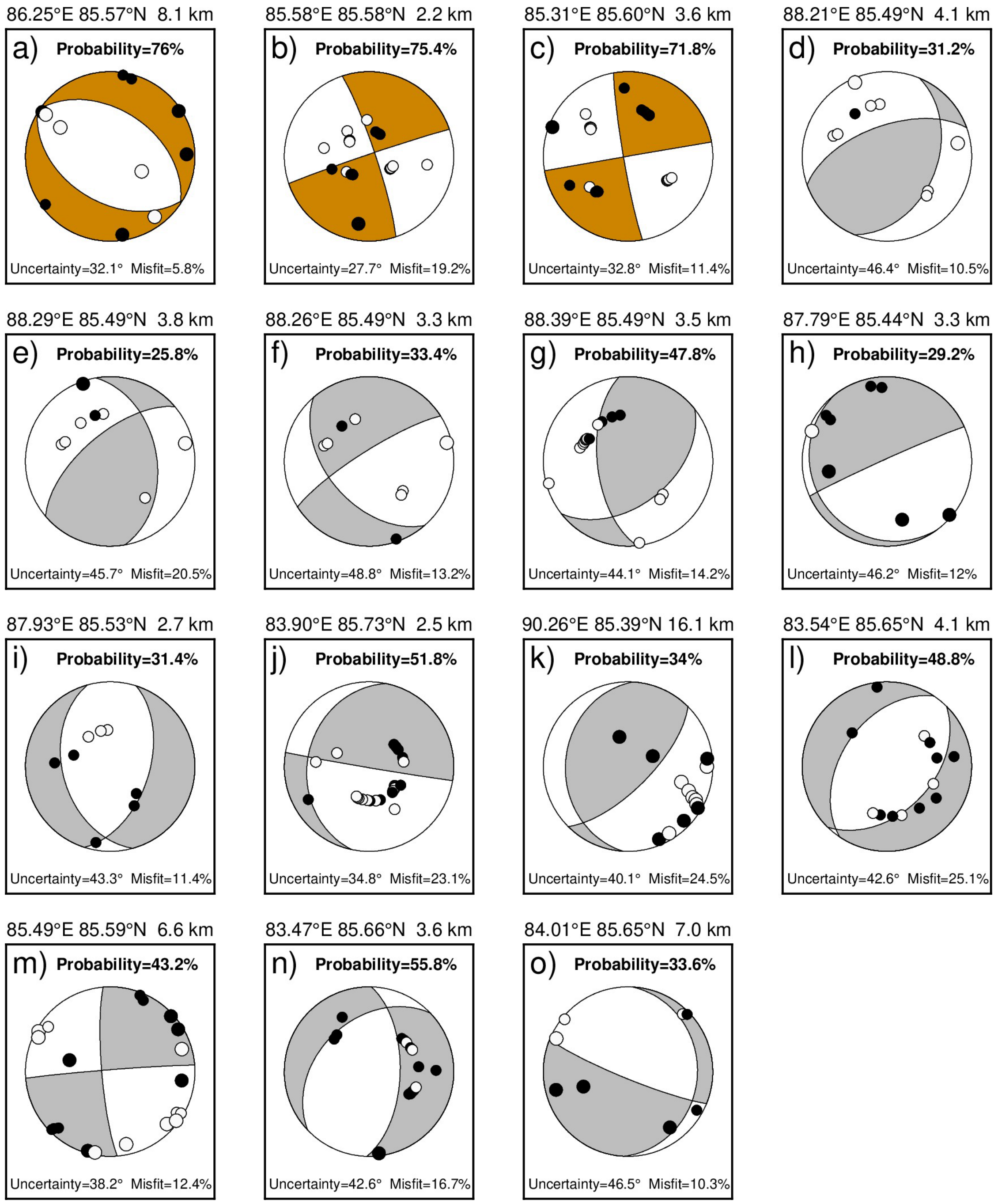

Figure S11. Focal mechanism solutions using P-wave first-motion arrivals. The earthquake hypocenters are shown at the top. Solution probability, fault plane uncertainties, and misfit are also provided. The brown ones show the three best solutions following quality **B**, under the HASH criteria [14].

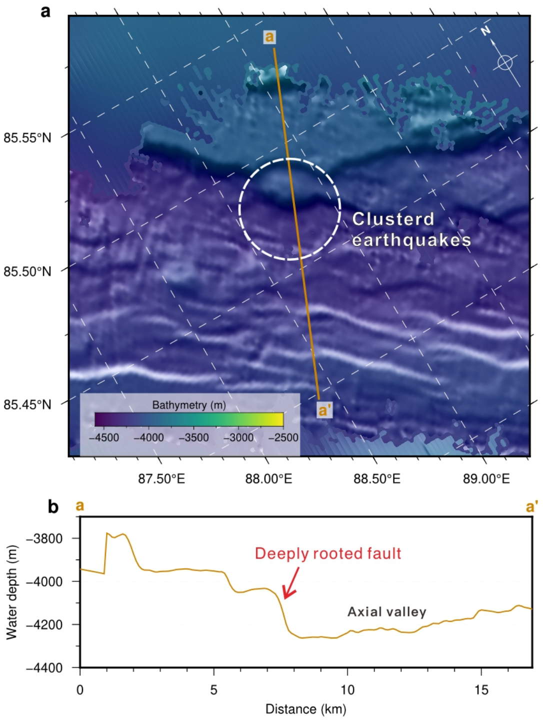

Figure S12. (**a**) Bathymetry of the 88°E segment end. The dashed circle shows the area of the clustered seismicity obtained in this study. (**b**) Water depth cross-section across the axial valley (profile location shown in **a**). The inferred deep fault exhibits a steep dip, with no rollover to a shallower angle.

Supplementary Tables

Table S1. The calculated focal mechanism solutions as shown in Figure S11.

No.	Longitude (°)	Latitude (°)	Depth (km)	Mechanism solution			RMS uncertainty		Polarity numbers	Average misfit	Mechanism probability (%)	Station distribution ratio (%)	Quality grade
				strike	dip	rake	fault plane	auxiliary plane					
1	85.5668	86.2490	8.07	120.9	45.1	-92.3	32.1	31.5	15	5.8	76	63.2	B
2	85.5847	85.5798	2.24	251.3	88.1	173.8	27.7	29	24	19.2	75.4	74.8	B
3	85.5985	85.3098	3.56	260.2	89.8	-175.6	32.8	31.8	27	11.4	71.8	85.6	B
4	85.4870	88.2123	4.12	250.3	60.3	118.7	46.4	45.1	10	10.5	31.2	77.9	D
5	85.4900	88.2892	3.76	229.7	63.8	127	51.7	45.7	8	20.5	25.8	74.2	D
6	85.4893	88.2568	3.26	237.6	80.2	-138.6	48.8	42	10	13.2	33.4	69.8	D
7	85.4890	88.3885	3.46	51.9	47.2	137.4	44.1	28.6	18	14.2	47.8	63.1	D
8	85.4392	87.7927	3.27	245.8	87.5	-101.3	46.2	44.9	9	12	29.2	68.7	D
9	85.5263	87.9287	2.74	17.8	51.9	-68	53	43.3	10	11.4	31.4	69.4	D
10	85.7298	83.9027	2.46	99.6	88.9	118	34.8	38.8	30	23.1	51.8	73.8	D
11	85.3875	90.2613	16.13	183.3	27	51.6	40.1	42.8	15	24.5	34	86.2	D
12	85.6477	83.5385	4.12	213.8	45.1	-97.6	42.6	38.2	14	25.1	48.8	62.8	D
13	85.5900	85.4912	6.57	265.5	88.2	-171.9	38.2	37	23	12.4	43.2	78.3	D
14	85.6592	83.4740	3.65	1.5	59.9	-116.5	42.6	34	20	16.7	55.8	63.8	D
15	85.6472	84.0052	6.98	112.2	83.4	-108.1	46.5	42.3	9	10.3	33.6	59	D

Table S2. The maximum depth of earthquakes versus full spreading rates at slow- and ultraslow-spreading Mid-Ocean Ridges. $D1_{max}$ and $D2_{max}$ indicate the maximum depth limited by several earthquakes and the deepest earthquake, respectively. Lat=Latitude; Lon=Longitude; MAR=Mid-Atlantic Ridge; SWIR=Southwest Indian Ridge; MCSC=Mid-Cayman Spreading Centre.

No	Name	Ridge centre	Area	Recording duration	Lat (°)	Lon (°)	Full rate (mm/yr)	Shallowest (km)	$D1_{max}$ (km)*	$D2_{max}$ (km)*
**1**	13°E -14°E (Oblique super-segment) [5]	SWIR	Indian	11 months	-52.38	13.50	7.8	1	16	17
**2**	Loki's Castle hydrothermal vent [17]	Knipovich Ridge	Arctic	11 months	73.6	8.1	13	1	7	8
**3**	85°E Volcanoes [18]	Gakkel Ridge	Arctic	16 days	85.00	85.00	10.0	1	16	23
**4**	85°E Volcanoes, this study	Gakkel Ridge	Arctic	22 days	85.00	85.00	10.0	0.5	9.7	13.6
**5**	Segment 1 [19]	SWIR	Indian	21 days	-25.70	69.80	12.6	0	10	10
**6**	Lena Trough [20]	Fram Strait	Arctic	15 days	81.00	-5.00	12.8	N	N	14
**7**	Segment 27 [21]	SWIR	Indian	21 days	-37.66	50.45	14.2	3	6	8
**8**	Segment 7 [22]	SWIR	Indian	11 months	-27.58	65.95	14.2	5	12	13
**9**	SWIR 64°30'E [23]	SWIR	Indian	21 days	-27.85	64.50	14.5	0	14	15
**10**	Logachev Seamount [24,25]	Knipovich Ridge	Arctic	10 days	76.50	7.20	14.5	2	6	12
**11**	Logachev Seamount-Amagmatic [24,25]	Knipovich Ridge	Arctic	10 days	76.20	7.20	14.5	7	16.5	20
**12**	Segment 28 [26]	SWIR	Indian	9 months	-37.72	49.70	14.6	2	13	15
**13**	Segment 28 [21]	SWIR	Indian	21 days	-37.72	49.70	14.6	0	16	20
**14**	Mount Dent [5]	MCSC	Caribbean	16 days	18.40	-81.75	15.0	1	7.5	9.5

No	Name	Ridge centre	Area	Recording duration	Lat (°)	Lon (°)	Full rate (mm/yr)	Shallowest (km)	$D1_{max}$ (km)*	$D2_{max}$ (km)*
**15**	Reykjanes Ridge [27]	MAR-Iceland	Atlantic	35 days	62.45	-25.80	20.0	0	7.5	12.5
**16**	Lucky strike [28]	MAR	Atlantic	24 months	37.33	-32.30	20.3	1.5	3	3.3
**17**	Lucky strike [29]	MAR	Atlantic	6 days	37.33	-32.30	20.3	1.5	6	6.5
**18**	35°N-West [30]	MAR	Atlantic	43 days	35.20	-36.50	20.6	0	4	4.5
**19**	Rainbow Massif [31]	MAR	Atlantic	9 months	36.20	-33.90	21.5	0	7.5	8
**20**	35°N-East [32]	MAR	Atlantic	43 days	35.10	-35.20	22.2	1	9	14
**21**	29°N [33]	MAR	Atlantic	41 days	29.20	-43.20	22.8	2.5	7.5	8
**22**	23°N [34,35]	MAR	Atlantic	10 days	23.50	-45.00	23.0	0.9	8	8
**23**	Logatchev Massif [36]	MAR	Atlantic	67 days	14.45	-45.00	24.0	1.5	5.5	7
**24**	26°N TAG [37]	MAR	Atlantic	8 months	26.10	44.85	24.2	0	7	8
**25**	26°N TAG [38]	MAR	Atlantic	23 days	26.10	44.85	24.2	2	7	8
**26**	13°20′N OCC [39,40]	MAR	Atlantic	10 days	13.33	-44.90	25.4	3	12	15
**27**	13°30′N OCC [39,40]	MAR	Atlantic	198 days	13.50	-44.85	25.4	4	10	12
**28**	5°S [41]	MAR	Atlantic	10 days	-5.20	-11.65	32.0	0	7	8
**29**	0°6′S[15]	MAR	Atlantic	21 days	-0.15	-16.45	32.0	1.5	16	18.5
**30**	7°12′S [36]	MAR	Atlantic	13 days	-7.20	-13.20	32.0	3	6	7
**31**	7°56′S [36]	MAR	Atlantic	24 days	-7.80	-13.40	32.0	2	4	7

Reference list

1. Ding W, Niu X, Zhang T *et al.* Submarine wide-angle seismic experiments in the High Arctic: The JASMInE Expedition in the slowest spreading Gakkel Ridge. *Geosystems Geoenvironment* 2022;**1**:100076.

2. Niksejel A, Zhang M. OBSTransformer: a deep-learning seismic phase picker for OBS data using automated labelling and transfer learning. *Geophys J Int* 2024;**237**:485–505.

3. Mousavi SM, Ellsworth WL, Zhu W *et al.* Earthquake transformer—an attentive deep-learning model for simultaneous earthquake detection and phase picking. *Nat Commun* 2020;**11**:3952.

4. Zhang T, Li J, Niu X *et al.* Highly variable magmatic accretion at the ultraslow-spreading Gakkel Ridge. *Nature* 2024;**633**:109–13.

5. Grevemeyer I, Hayman NW, Lange D *et al.* Constraining the maximum depth of brittle deformation at slow- and ultraslow-spreading ridges using microseismicity. *Geology* 2019;**47**:1069–73.

6. Yu Z, Singh SC, Gregory EPM *et al.* Semibrittle seismic deformation in high-temperature mantle mylonite shear zone along the Romanche transform fault. *Sci Adv* 2021;**7**:eabf3388.

7. Lomax A, Virieux J, Volant P *et al.* Probabilistic earthquake location in 3D and layered models. *Advances in Seismic Event Location*. Springer, 2000, 101–34.

8. Waldhauser F, Ellsworth WL. A double-difference earthquake location algorithm: Method and application to the northern Hayward fault, California. *Bull Seismol Soc Am* 2000;**90**:1353–68.

9. Efron B, Tibshirani R. Statistical Data Analysis in the Computer Age. *Science* 1991;**253**:390–5.

10. Efron B, Gong G. A Leisurely Look at the Bootstrap, the Jackknife, and Cross-Validation. *Am Stat* 1983;**37**:36–48.

11. Hutton L, Boore DM. The ML scale in southern California. *Bull Seismol Soc Am*

1987;**77**:2074–94.

12. Goldstein P, Snoke A. SAC Availability for the IRIS Community. *Inc Res Inst Seismol Newsl* 2005:UCRL-JRNL-211140.

13. Skoumal RJ, Hardebeck JL, Shearer PM. SKHASH: A Python Package for Computing Earthquake Focal Mechanisms. *Seismol Res Lett* 2024;**95**:2519–26.

14. Hardebeck JL, Shearer PM. A new method for determining first-motion focal mechanisms. *Bull Seismol Soc Am* 2002;**92**:2264–76.

15. Yu Z, Singh SC, Hamelin C *et al.* Deep mantle earthquakes linked to CO2 degassing at the Mid-Atlantic Ridge. *Nat Commun* 2025;**16**:563.

16. Christeson GL, Goff JA, Reece RS. Synthesis of Oceanic Crustal Structure From Two-Dimensional Seismic Profiles. *Rev Geophys* 2019;**57**:504–29.

17. Pilot M, Lien MJ, Schlindwein V *et al.* Microseismicity Around Loki's Castle Hydrothermal Vent Field Reveals the Early Stages of Detachment Faulting at the Mohns-Knipovich Ridge Intersection. *Geochem Geophys Geosystems* 2024;**25**:e2024GC011732.

18. Korger EIM, Schlindwein V. Seismicity and structure of the 85°E volcanic complex at the ultraslow spreading Gakkel Ridge from local earthquake tomography. *Geophys J Int* 2014;**196**:539–51.

19. Katsumata K, Sato T, Kasahara J *et al.* Microearthquake seismicity and focal mechanisms at the Rodriguez Triple Junction in the Indian Ocean using ocean bottom seismometers. *J Geophys Res Solid Earth* 2001;**106**:30689–99.

20. Läderach C, Schlindwein V, Schenke H-W *et al.* Seismicity and active tectonic processes in the ultra-slow spreading Lena Trough, Arctic Ocean: Seismicity of Lena Trough. *Geophys J Int* 2011;**184**:1354–70.

21. Yu Z, Li J, Niu X *et al.* Lithospheric structure and tectonic processes constrained by microearthquake activity at the central ultraslow-spreading Southwest Indian Ridge (49.2° to 50.8°E). *J Geophys Res Solid Earth* 2018;**123**:6247–62.

22. Meier M, Schlindwein V. First In Situ Seismic Record of Spreading Events at the

Ultraslow Spreading Southwest Indian Ridge. *Geophys Res Lett* 2018;**45**:10,360-10,368.

23. Chen J, Crawford WC, Cannat M. Microseismicity and lithosphere thickness at a nearly-amagmatic oceanic detachment fault system. *Nat Commun* 2023;**14**:430.

24. Schlindwein V, Demuth A, Geissler WH *et al.* Seismic gap beneath Logachev Seamount: Indicator for melt focusing at an ultraslow mid-ocean ridge? *Geophys Res Lett* 2013;**40**:1703–7.

25. Meier M, Schlindwein V, Scholz J *et al.* Segment-Scale Seismicity of the Ultraslow Spreading Knipovich Ridge. *Geochem Geophys Geosystems* 2021;**22**:e2020GC009375.

26. Tao C, Seyfried WE, Lowell RP *et al.* Deep high-temperature hydrothermal circulation in a detachment faulting system on the ultra-slow spreading ridge. *Nat Commun* 2020;**11**:1300.

27. Mochizuki M, Brandsdóttir B, Shiobara H *et al.* Detailed distribution of microearthquakes along the northern Reykjanes Ridge, off SW-Iceland. *Geophys Res Lett* 2000;**27**:1945–8.

28. Crawford WC, Rai A, Singh SC *et al.* Hydrothermal seismicity beneath the summit of Lucky Strike volcano, Mid-Atlantic Ridge. *Earth Planet Sci Lett* 2013;**373**:118–28.

29. Dusunur D, Escartín J, Combier V *et al.* Seismological constraints on the thermal structure along the Lucky Strike segment (Mid-Atlantic Ridge) and interaction of tectonic and magmatic processes around the magma chamber. *Mar Geophys Res* 2009;**30**:105–20.

30. Barclay AH. Shear wave splitting and crustal anisotropy at the Mid-Atlantic Ridge, 35°N. *J Geophys Res* 2003;**108**:2378.

31. Horning G, Sohn RA, Canales JP *et al.* Local Seismicity of the Rainbow Massif on the Mid-Atlantic Ridge. *J Geophys Res Solid Earth* 2018;**123**:1615–30.

32. Cessaro RK, Hussong DM. Transform seismicity at the intersection of the oceanographer fracture zone and the Mid-Atlantic Ridge. *J Geophys Res* 1986;**91**:4839–53.

33. Wolfe CJ, Purdy GM, Toomey DR *et al.* Microearthquake characteristics and crustal velocity structure at 29°N on the Mid-Atlantic Ridge: The architecture of a slow spreading segment. *J Geophys Res Solid Earth* 1995;**100**:24449–72.

34. Toomey DR, Solomon SC, Purdy GM. Microearthquakes beneath Median Valley of Mid-Atlantic Ridge near 23°N: Tomography and tectonics. *J Geophys Res* 1988;**93**:9093–112.

35. Toomey DR, Solomon SC, Purdy GM *et al.* Microearthquakes beneath the Median Valley of the Mid-Atlantic Ridge near 23°N: Hypocenters and focal mechanisms. *J Geophys Res Solid Earth* 1985;**90**:5443–58.

36. Grevemeyer I, Reston TJ, Moeller S. Microseismicity of the Mid-Atlantic Ridge at 7°S-8°15′S and at the Logatchev Massif oceanic core complex at 14°40′N-14°50′N. *Geochem Geophys Geosystems* 2013;**14**:3532–54.

37. deMartin BJ, Sohn RA, Pablo Canales J *et al.* Kinematics and geometry of active detachment faulting beneath the Trans-Atlantic Geotraverse (TAG) hydrothermal field on the Mid-Atlantic Ridge. *Geology* 2007;**35**:711–4.

38. Kong LSL, Solomon SC, Purdy GM. Microearthquake Characteristics of a Mid-Ocean Ridge along-axis high. *J Geophys Res Solid Earth* 1992;**97**:1659–85.

39. Parnell-Turner R, Sohn RA, Peirce C *et al.* Seismicity trends and detachment fault structure at 13°N, Mid-Atlantic Ridge. *Geology* 2021;**49**:320–4.

40. Parnell-Turner R, Sohn RA, Peirce C *et al.* Oceanic detachment faults generate compression in extension. *Geology* 2017;**45**:923–6.

41. Tilmann F, Flueh E, Planert L *et al.* Microearthquake seismicity of the Mid-Atlantic Ridge at 5°S: A view of tectonic extension. *J Geophys Res Solid Earth* 2004;**109**:B06102.
